# The Association Between Long-Term Acenocoumarol Treatment and Vitamin D Deficiency

**DOI:** 10.3389/fendo.2018.00226

**Published:** 2018-05-04

**Authors:** Jolanta Sawicka-Powierza, Jerzy Konstantynowicz, Ewa Jablonska, Beata Zelazowska-Rutkowska, Wojciech Jelski, Pawel Abramowicz, Caroline Sasinowski, Slawomir Chlabicz

**Affiliations:** ^1^Department of Family Medicine, Medical University of Bialystok, Bialystok, Poland; ^2^Department of Haematology, Medical University of Bialystok, Bialystok, Poland; ^3^Department of Pediatrics, Rheumatology, Immunology, and Metabolic Bone Diseases, Medical University of Bialystok, Bialystok, Poland; ^4^Department of Immunology, Medical University of Bialystok, Bialystok, Poland; ^5^Department of Pediatric Laboratory Diagnostics, Medical University of Bialystok, Bialystok, Poland; ^6^Department of Biochemical Diagnostics, Medical University of Bialystok, Bialystok, Poland; ^7^University Clinical Hospital, Medical University of Bialystok, Bialystok, Poland

**Keywords:** acenocoumarol, vitamin D deficiency, vitamin K antagonist, chronic disease, long-term treatment

## Abstract

**Objective:**

Both vitamin D and K2 are involved in a number of metabolic processes, including bone metabolism; however, associations between the vitamins are not fully understood. The aim of the study was to evaluate serum concentrations of 25-hydroxyvitamin D [25(OH)D] in adult patients receiving long-term acenocoumarol (AC) treatment.

**Participants and methods:**

In this cross-sectional study, 58 Caucasian patients (31 women, 27 men) with a median age of 65 years receiving long-term AC therapy were evaluated and compared with 35 age- and gender-matched healthy controls. The AC treatment was used due to recurrent venous thromboembolism (34.5%), atrial fibrillation (31%), or mechanical heart valve prostheses (34.5%). Medical records and a questionnaire were used to obtain information about chronic diseases, smoking habits, and the duration of therapy and weekly dose of AC. Anthropometric measurements were performed, and serum concentration of 25(OH)D and total alkaline phosphatase (ALP) activity were measured.

**Results:**

Among the 58 patients receiving long-term AC treatment, a high proportion (46.6%) demonstrated significant vitamin D deficiency with concentrations of 25(OH)D lower than 20 ng/mL. The median concentration of 25(OH)D in subjects receiving AC was significantly lower compared to the control group [20.4 (17.4; 26.1) vs. 28.2 (24; 32.7); *p* < 0.001]. No differences were found between women and men receiving AC therapy. In patients receiving AC, a negative correlation was found between the concentration of 25(OH)D and the weekly dose of AC (*r* = −0.337, *p* = 0.01). Patients with concentrations of 25(OH)D < 20 ng/mL were found to have a significantly higher median dose of AC, compared to those with concentrations of 25(OH)D ≥ 20 ng/mL [21 (17; 31) vs. 17 (12; 28); *p* = 0.045].

**Conclusion:**

In conclusion, treatment with AC is associated with low 25-hydroxyvitamin D levels, although the path leading to this phenomenon is not entirely clear. Long-term administration of AC in adults may increase the risk of chronic vitamin D deficiency, thus, effective supplementation of vitamin D in these individuals needs careful consideration.

## Introduction

Vitamin D synthesized in the skin or obtained from the diet is biologically inactive. Enzymatic conversion in the liver and kidney is required for its activation. Cholecalciferol [vitamin D(3)], inherently present in animals, is converted to calcifediol (25-hydroxycholecalciferol) in the liver, whereas ergocalciferol [vitamin D(2)], naturally found in plants, is converted to 25-hydroxyergocalciferol. These two vitamin D metabolites [called 25-hydroxyvitamin D or 25(OH)D] are measured in serum to determine vitamin D status (1). 25(OH)D is further hydroxylated by the kidneys to form calcitriol (1,25-dihydroxycholecalciferol), the biologically active form of vitamin D (2). The presence of 1-α-hydroxylase has been confirmed in bone tissue; hence bone itself is regarded as a source of the active form of vitamin D (3). Vitamin D exerts direct effects on various populations of bone cells, and functions indirectly by regulating both calcium-phosphate homeostasis (4) and the expression of parathormone (5). Vitamin D stimulates proliferation and differentiation of osteoblasts and osteoclasts (6), regulates the expression of numerous genes in the bone cell population (7), plays a role in the synthesis of key proteins secreted by osteoblasts, and inhibits apoptosis of osteoblasts (8). Vitamin D also regulates the expression of proteins involved in intestinal calcium absorption and calcium reabsorption by renal tubules, which ensures adequate mineralization of osteoid. Thus, bone metabolism largely depends on the biological action and adequate levels of vitamin D.

Experimental and observational studies, as well as clinical trials, indicate that insufficient intake of vitamin K2 and/or long-term treatment with vitamin K antagonists (VKAs) are related to bone metabolism disorders and arterial calcification (9–13). The specific mechanism of how vitamin K2 impacts bone metabolism has yet to be fully explained and understood. It is known that dietary calcium is absorbed from the digestive tract with the participation of vitamin D, while both vitamins, D and K2, are responsible for the production of the active form of osteocalcin, an integral protein involved in the synthesis of bone matrix and binding of calcium ions (14). Interestingly enough, an excess of calcium intake, particularly derived from supplementation, when combined with vitamin K2 deficiency, may result in the deposition of calcium in blood vessels (arterial calcification) and may increase the risk of soft tissue calcification (15). VKAs, including acenocoumarol (AC), suppress the synthesis of vitamin K-dependent proteins. Instead, alternative pathways are initiated resulting in the formation of undercarboxylated Gla proteins. In the bone, this results in the synthesis of the functionally inactive form of osteocalcin (16). The information above confirms the existence of links between vitamins D, K2, and bone metabolism, and could explain the potential pathogenic role of VKAs in impaired bone metabolism. For this reason, the possible connection between the use of VKA and vitamin D status may also be worth investigating in the context of chronic diseases and treatment.

According to the current guidelines, hypovitaminosis D is diagnosed by measuring serum concentration of 25-hydroxyvitamin D {[25(OH)D]; calcidiol}, the most commonly accepted indicator of vitamin D status. Levels of 25(OH)D below 20 ng/mL (50 nmol/L) are indicative of a deficiency and levels of 20–30 ng/mL (50–75 nmol/L) indicate an insufficiency, although the definition of deficiency and insufficiency remain a subject of discussion (17, 18). Several negative skeletal effects and bone mineral disorders may be attributed to both vitamin D deficiency and suboptimal vitamin K2 levels. It has been reported that vitamin K2, combined with vitamin D, increases bone mineral density more efficiently than vitamin K2 alone (19). Some study has also shown that vitamin K2 supplementation improves hip bone geometry and bone strength indices among postmenopausal women (20). Given that the effects of vitamin K2 may be generally beneficial for bone health, the suppression of vitamin K2 by VKA may potentially lead to impairment of bone metabolism and, therefore, a deteriorated skeletal status. However, as there are still conflicting reports and inconsistent data, the question arises whether long-term use of VKAs with coexisting vitamin D deficiency may have clinical implications or be a risk for patients.

The objective of the study was to evaluate vitamin D status expressed as serum concentration of 25-hydroxyvitamin D [25(OH)D] in adult patients receiving long-term AC treatment.

## Materials and Methods

### Participants and Study Protocol

The cross-sectional study was conducted among adult patients treated with AC and healthy individuals recruited from the population of 5,834 people of a primary care practice. Participants’ data were initially retrieved from an electronic database and medical records of the primary care facility taking part in the study. The obtained data contained information about age, gender, indications for anticoagulant treatment, and type of administered medication. Patients were previously qualified for AC prophylaxis due to recurrent venous thromboembolism, atrial fibrillation, or mechanical heart valve prostheses. The age- and gender-matched control group was recruited from the healthy population of the primary care practice using a random numbers table. The inclusion criteria consisted of written informed consent and duration of AC treatment longer than 3 months. Based on a questionnaire and medical records, participants with chronic diseases (i.e., chronic renal, gastrointestinal, liver, and endocrine diseases), those receiving treatment affecting vitamin D status/metabolism (i.e., anticonvulsants, systemic glucocorticosteroids), and those taking vitamin D and/or K2 supplements, were excluded from the study.

Seventy-two patients treated with AC, and 70 healthy subjects (controls) were invited to the study. Fourteen patients receiving AC, as well as 22 controls, did not meet all eligibility criteria and were excluded. Additionally, 13 healthy subjects refused to participate. Ultimately, 58 Caucasian patients (27 males, 31 females) with median age 65 years receiving long-term AC treatment for recurrent venous thromboembolism (*n* = 20; 34.5%), atrial fibrillation (*n* = 18; 31%), and mechanical heart valve prostheses (*n* = 20; 34.5%) were enrolled into the study. Thirty-five healthy subjects (16 males, 19 females) with median age 61 years constituted the control group. Evaluation of all participants taking part in the study was done exclusively on the basis of medical records and a questionnaire. All participants were screened for chronic diseases and therapies known to affect vitamin D status. None of the participants had been supplemented with vitamin D and/or fish oil or vitamin K prior to and during the study. Study subjects were asked about the duration and weekly dose of AC treatment, smoking habits (past and current), time of menopause (if applicable), and the time spent outdoors. The majority of the participants (66 individuals; 71%) were people working in their profession, while others in the sample were not older than 80 years old. Furthermore, neither the patients receiving AC nor any of the controls had conditions that would have made ambulating difficult. Finally, time spent outdoors was comparable among subjects included in the patient and the control group.

The study protocol and procedures were approved by the Ethics Committee of the Medical University of Bialystok (No R-I-002/88/2013).

### Measurements

Blood was collected from the study subjects in the winter season (between January and March) to avoid seasonal variations of vitamin D concentration. Blood samples were collected before 9:00 a.m. on the day of the interview after overnight fasting. All samples were centrifuged within 60 min from blood draw and sera were stored at −70°C until measurement.

The Elecsys Vitamin D(3) (25-OH) immunoassay was used for the quantitative determination of 25-hyroxyvitamin D(3) on cobas e immunoassay analyzer (Roche Diagnostics, Indianapolis, IN, USA). Activity of alkaline phosphatase was measured by colorimetric method (Abbott Laboratories, Abbott Park, Illinois, USA) with para-nitrophenyl phosphate as substrate. The reaction was monitored at 404 nm on an Architect ci8200 analyzer (Architect System, Abbott Laboratories, IL, USA).

Standing height and body weight were measured using standard anthropometric methods (wall-mounted stadiometer, electronic scale; Seca, Germany) and body mass index (BMI) was calculated using the standard formula.

### Statistical Analyses

Statistical analyses were performed using the Statistical Package for the Social Sciences (SPSS), version 11.5. The Shapiro–Wilk test was used to examine normal distribution. Since the data were not normally distributed, the results were presented as a median, 25th and 75th percentile. Nonparametric *t*-test and Mann–Whitney *U* test were applied for comparison between groups. Chi^2^ test was used for qualitative data. Correlations between parameters were evaluated with Spearman’s rank correlation coefficient. For the calculations, *p* < 0.05 was adopted as the level of statistical significance.

## Results

No differences in terms of age, gender, BMI, time elapsed, since menopause (females), smoking habits, and ALP activity were found between subjects receiving AC and the control group. All participants had ALP activity within the normal range (normal values: 37–110 IU/L). The median concentration of 25(OH)D in patients treated with AC was significantly lower compared to controls (*p* < 0.001) (Table 1). Anthropometric and biochemical parameters did not significantly differ between the groups of patients receiving AC prophylaxis for recurrent venous thromboembolism, atrial fibrillation, and mechanical heart valve prostheses. No differences between women and men receiving AC were found with regards to the studied variables.

**Table 1:** Basic characteristics of study participants.

Characteristics	Subjects receiving acenocoumarol	Control subjects	*p*
Number, *n*	58	35	
Age, years	65 (59; 73)	61 (59; 72)	NS
Gender (male/female), *n* (%)	27 (46.6)/31 (53.4)	16 (45.7)/19 (54.3)	NS
Body mass index, kg/m^2^	28.1 (24.9; 31.3)	27.3 (24.4; 29.4)	NS
Post-menopause, years	15 (8.8; 23.3)	9 (6; 23)	NS
Smokers/non-smokers, *n* (%)	11 (19)/47 (81)	4 (11.4)/31 (88.6)	NS
Duration of AC treatment, years	8 (6.8; 12.3)	0	<0.001
Weekly dose of AC, mg	19 (14; 28)	0	<0.001
Serum 25(OH)D, ng/mL	20.4 (17.4; 26.1)	28.2 (24; 32.7)	<0.001
ALP, IU/L	61 (54.3; 75)	54 (51; 68)	NS

*Results are shown as median and quartiles (Q1; Q3)*.

*To convert values for 25-hydroxyvitamin D to nmol/L, multiply by 2.5*.

**Figure 1:**
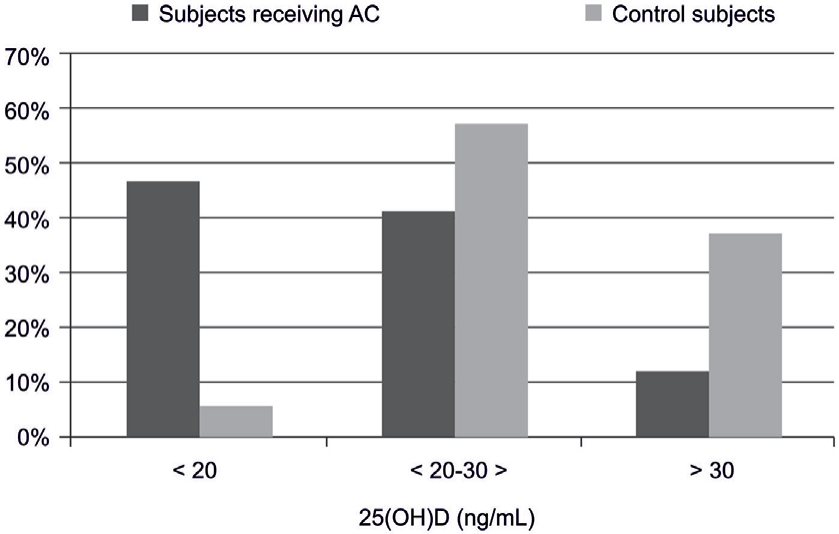
The significant difference in percent distribution of 25(OH)D levels in subjects receiving acenocoumarol treatment and controls (*n* = 93).

Figure 1 presents the proportion of subjects with 25(OH)D level below 20 ng/mL, between 20 and 30 ng/mL, and those above 30 ng/mL. It was found that 27 (46.5%) individuals receiving AC had vitamin D deficiency, i.e., 25(OH)D levels below 20 ng/mL. Such deficiency was observed in only 2 (5.7%) control subjects. Levels of 25(OH)D between 20 and 30 ng/mL were found in 24 (41.4%) subjects treated with AC, and in 20 (57.1%) control subjects. Normal 25(OH)D values, defined as higher than 30 ng/mL, were found only in 7 (12.1%) patients receiving AC treatment and in 13 (37.2%) controls. Patients receiving AC had a significantly larger proportion of decreased 25(OH)D concentration compared to controls (*p* < 0.001).

**Figure 2:**
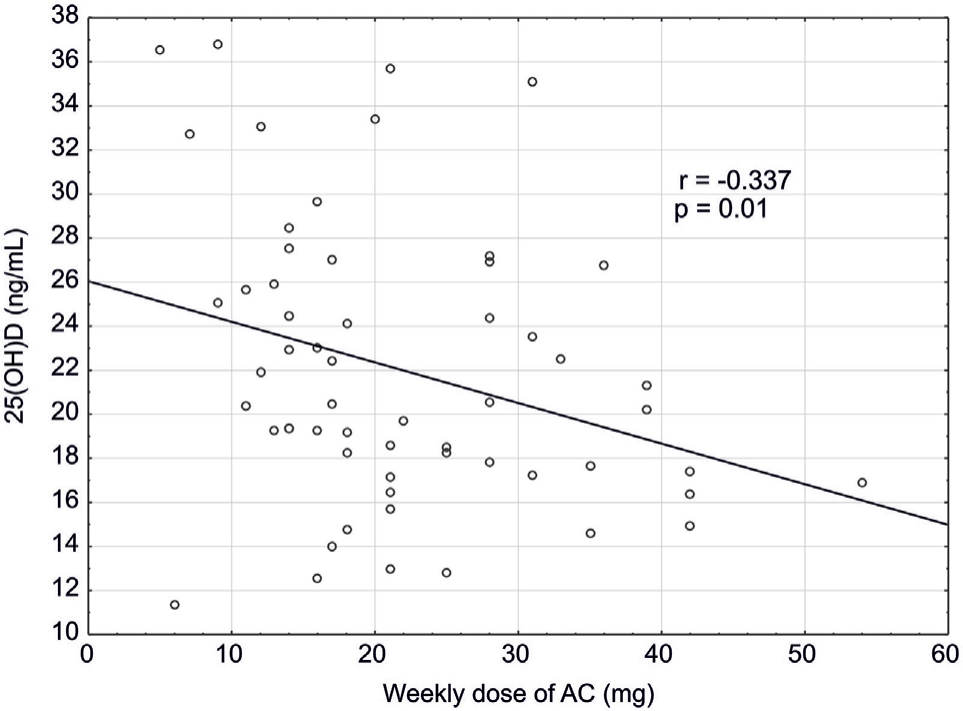
Correlation between concentration of 25(OH)D (ng/mL) and a weekly dose of acenocoumarol (mg) in studied patients (*n* = 58).

Among patients receiving AC treatment, a negative correlation was found between the weekly dose of AC and serum concentration of 25(OH)D (*r* = −0.337; *p* = 0.01) (Figure 2), and between the weekly dose of AC and age (*r* = −0.352; *p* = 0.007). Also, a positive correlation between ALP activity and age was observed (*r* = 0.272; *p* = 0.039).

In the control group, a negative correlation was found between 25(OH)D level and age, and between 25(OH)D and the time, since menopause (*r* = −0.395; *p* = 0.019 and *r* = −0.717; *p* = 0.003, respectively). ALP activities positively correlated with age (*r* = 0.403; *p* = 0.017).

Among patients receiving AC, the studied parameters were compared between two groups of patients; those with a threshold concentration of 25 (OH)D < 20 ng/mL indicating a deficiency, and the group of patients with a concentration of 25 (OH)D ≥ 20 ng/mL. We did not find statistically significant differences in median age, BMI, time from last menstruation, the duration of treatment, and ALP activity between the groups. Both studied groups differed significantly in the median values of weekly dose of AC (*p* = 0.045), the median of 25(OH)D concentration (*p* < 0.001), and proportion of female patients (*p* = 0.015) (Table 2).

**Table 2:** Comparison of studied parameters, depending on 25(OH)D serum concentration in subjects treated with acenocoumarol (AC) (the cut-off value set at 20 ng/mL was used to define the deficiency level).

Characteristics	Subjects receiving AC with 25(OH)D < 20 ng/mL	Subjects receiving AC with 25(OH)D ≥ 20 ng/mL	*p*
Number, *n*	27	31	
Age, years	65 (60; 73)	65 (59; 73)	NS
Gender (males/females), *n* (%)	8 (29.6)/19 (70.4)	19 (61.3)/12 (38.7)	0.015
Body mass index, kg/m^2^	27.9 (24.8; 31.2)	28.4 (24.9; 32)	NS
Post-menopause, years	15 (8.8; 24)	15 (8.5; 19.8)	NS
Smokers/non-smokers, *n* (%)	3 (11.1)/24 (88.9)	8 (25.8)/23 (74.2)	NS
Duration of AC treatment, years	8 (6; 10)	10 (7; 13)	NS
Weekly dose of AC, mg	21 (17; 31)	17 (12; 28)	0.045
Serum 25(OH)D, ng/mL	17.2 (14.8; 18.6)	25.6 (22.6; 29.6)	<0.001
ALP, IU/L	62 (58; 76)	59 (52; 74)	NS

*Results are shown as median and quartiles (Q1; Q3)*.

## Discussion

The significantly decreased concentration of 25(OH)D observed in men and women treated with AC may indicate a potential negative effect of this therapy on vitamin D status, and presumably on bone metabolism or possible prospective health outcomes. Our results demonstrated that a large proportion of subjects receiving AC had vitamin D deficiency with the concentrations below 20 ng/mL. Our results are consistent with the results of other cross-sectional studies confirming the low level of vitamin D in patients receiving VKAs. For example, lower 25(OH)D levels in those taking VKAs were also seen in four cross-sectional studies performed among 514 Dutch females (21), 7,553 German males (22), 783 Netherlands geriatric outpatients (23), and 48 Greek children (24). On the contrary, in two other cross-sectional studies (*n* = 127, *n* = 116, respectively) and one prospective cohort study (*n* = 167), no such associations were observed (25–27). Different recruitment methods, designs and methodologies, the influence of still unknown factors, or different duration of treatment may have caused such differences in results across studies presented above.

To the best of our knowledge, our study is the first to demonstrate the inverse association between the dose of AC and vitamin D levels, so there is limited possibility to compare our results with others. The possible relationship between the dose of VKA and the level of vitamin D may be confirmed by the recently published study on the treatment of vitamin D deficiency in patients with venous thromboembolism. The study showed that treatment of vitamin D deficiency in patients with venous thromboembolism, resulted in the control of the international normalized ratio with the lower doses of warfarin. This observation was the first clinical report of an enhancement of the anticoagulant effect of warfarin by vitamin D supplementation (28).

Some common mechanisms or overlapping pathways may be involved in the regulation of the biological effects of these two vitamins. Wang et al. have identified a novel CYP3A4-dependent pathway, of 4-hydroxylation of 25-hydroxyvitamin D(3), the induction of which may contribute to drug-induced vitamin D deficiency. These results suggested that the CYP3A4-dependent metabolism of vitamin D may be important for the regulation of vitamin D(3) levels *in vivo* and in the etiology of drug-induced osteomalacia (29). It is well known, that oxidative metabolism of isomer R-AC is catalyzed by several members of the cytochrome P-450 family in the liver, including CYP3A4. Those data, along with our observations, suggest a mechanism that leads to decreased vitamin D levels in patients on long-term VKA treatment. The interplay between vitamins K and D is apparent, for instance, in the form of osteocalcin, being essential for the formation of hydroxyapatite crystals in bone tissue (30, 31). Vitamin K2 can act not only through vitamin K-dependent proteins, but is also able to directly impact the gene expression by binding to steroid and xenobiotic receptors (32). Furthermore, vitamin K and D overlap metabolically at the cellular level. The cyclic oxidation and reduction of vitamin K is a source of electron transfer for antioxidant power to protect living cells against oxidative stress (33). Similarly, 1,25(OH)D has anti-oxidative capacity, as demonstrated in animal studies (34). It can, therefore, be concluded that vitamins K and D interact and stimulate each other’s metabolism. The activity of vitamin D metabolites can be regarded as a gatekeeper, controlling calcium absorption, while the activity of vitamin K2 can be seen as that of a traffic policeman, directing the calcium ions into the bone (35).

The major limitation of the study was the small sample size of recruited subjects, particularly of the control group (controls were not as numerous) and the observational design of the study, which did not allow us to draw conclusions about causality. Another limitation was the inability to assess concentration of parathormone and osteocalcin among participants. There was also the possibility that some subjects may have had lower vitamin D levels for reasons other than the treatment with AC. By way of example, individuals in poor physical condition are likely to spend more time indoors than healthy ones, thus are prone to inadequate natural ultraviolet B (UVB) exposure which could in turn lead to extremely limited skin synthesis of vitamin D, and decreased 25(OH)D levels. The majority of the participants in this study were people actively working in their profession and none of the participants had a condition that would have made ambulating difficult, thus, in fact, time spent outdoors did not essentially affect the results. Nevertheless, neither the methodological issues nor other factors could detract from the main observation in this report. Our study revealed that AC doses negatively correlated with 25(OH)D concentrations, suggesting a possible association between VKA therapy and decreased vitamin D status. We also found that the median of weekly dose of AC was significantly higher in patients with lower vitamin D levels compared to patients with higher vitamin D levels. Further, patients in poor general physical condition do not generally receive higher doses of AC. Since our study was conducted during the winter months, when sunlight exposure was extremely low (latitude 53–54^o^N), the potential impact of UVB on vitamin D status among studied participants may be regarded as irrelevant. Nevertheless, explanation of this phenomenon does require further study.

To summarize, the results obtained in this study may indicate that long-term therapy with AC may be related to low levels of vitamin D. Thus far, a large number of data have been published regarding global consequences of vitamin D deficiency in the context of human morbidity, pleiotropic capacity, multifactorial associations between medical therapies, and vitamin D metabolism, in a variety of populations and risk groups (4). The general recommendations for vitamin D supplementation in healthy adult populations, although undergoing a vivid debate, are well known and commonly accepted, however, the list of populations at risk of deficiency have not yet been fully determined (18, 36–39). Future intervention studies warrant explanation and understanding of the underlying mechanisms of our findings, and may presumably allow the determination of precise recommendations for vitamin D-deficient patients treated with VKAs. Our observations, discussed above, suggest that 25-hydroxyvitamin D concentrations should be routinely measured and monitored in all patients receiving specifically long-term treatment with VKAs.

In conclusion, treatment with AC is associated with lower levels of 25-hydroxyvitamin D. Long-term administration of AC in adults may potentiate the risk of chronic vitamin D deficiency, thus, effective well-coordinated supplementation of vitamin D in these individuals needs careful consideration.

## Ethics Statement

This study was carried out in accordance with the recommendations of the Declaration of Helsinki; written informed consent from all participants was obtained. Funded by Medical University of Bialystok. The Medical University of Bialystok in Poland approved the protocol (Approval: No. R-I-002/88/2013).

## Author Contributions

Design of the study: JS-P and JK. Data collection and analyses: JS-P, EJ, BZ-R, and WJ. Laboratory tests: BZ-R and WJ. Data evaluation: JS-P, JK, and EJ. Writing and preparation of the manuscript: JS-P, JK, EJ, and PA. Proof-reading and editing: JS-P, JK, EJ, and CS.

## Conflict of Interest Statement

The authors declare that the research was conducted in the absence of any commercial or financial relationships that could be construed as a potential conflict of interest. The reviewer EC and handling Editor declared their shared affiliation.

## Abbreviations

AC, acenocoumarol; ALP, serum total alkaline phosphatase; BMI, body mass index; NS, non significant; VKA, vitamin K antagonist; 25(OH)D, 25-hydroxyvitamin D.
